# Impact of chronic *Pseudomonas aeruginosa* infection on health-related quality of life in *Mycobacterium avium* complex lung disease

**DOI:** 10.1186/s12890-017-0544-x

**Published:** 2017-12-13

**Authors:** Hirofumi Kamata, Takanori Asakura, Shoji Suzuki, Ho Namkoong, Kazuma Yagi, Yohei Funatsu, Satoshi Okamori, Shunsuke Uno, Yoshifumi Uwamino, Hiroshi Fujiwara, Tomoyasu Nishimura, Makoto Ishii, Tomoko Betsuyaku, Naoki Hasegawa

**Affiliations:** 1 Division of Pulmonary Medicine, Department of Medicine, Keio University School of Medicine, 35 Shinanomachi, Shinjuku, Tokyo, Japan; 2 Japan Society for the Promotion of Science, Chiyoda, Tokyo, Japan; 3 Center for Infectious Diseases and Infection Control, Keio University School of Medicine, 35 Shinanomachi, Shinjuku, Tokyo, 160-8582 Japan; 4 Department of Laboratory Medicine, Keio University School of Medicine, 35 Shinanomachi, Shinjuku, Tokyo, Japan; 5 Health Center, Keio University, Shinjuku, Tokyo, Japan

**Keywords:** Colonisation, Noncystic fibrosis bronchiectasis (NCFBE), Health-related quality of life (HRQL), *Mycobacterium avium* Complex (MAC), Nontuberculous mycobacteria (NTM), *Pseudomonas aeruginosa*, St. George’s Respiratory Questionnaire (SGRQ), 36-item short-form health survey (SF-36)

## Abstract

**Background:**

In bronchiectasis patients, chronic *Pseudomonas aeruginosa* (PA) infection has been associated with worse health-related quality of life (HRQL), but little is known about *Mycobacterium avium* complex lung disease (MACLD) patients in this context. This study aimed to evaluate HRQL and investigate the impact of chronic PA infection in MACLD patients.

**Methods:**

This cross-sectional study was conducted using the Registry of Prospective Cohort Study including MACLD patients. The 36-item Short-Form health survey (SF-36) and St. George’s Respiratory Questionnaire (SGRQ) were administered to assess clinical outcomes. Clinical variables included treatment and sputum culture status, pulmonary function tests, cavitary lesions, and modified Reiff scores on high-resolution computed tomography.

**Results:**

The study included 244 MACLD patients (median age, 68 years; 196 women), 19 of whom had chronic PA infection. Modified Reiff score was higher in patients with chronic infection than in those without (*P* = 0.028). Regarding SF-36 scores, physical functioning subscale scores were significantly lower in patients with chronic infection (*P* = 0.029). Additionally, SGRQ symptoms, impact, and total scores were significantly higher in patients with chronic infection. During analysis of covariance comparisons, SGRQ symptoms and impact scores were significantly higher for patients with chronic infection (*P* = 0.043 and 0.021, respectively).

**Conclusions:**

MACLD patients with chronic PA infection exhibited significantly higher SGRQ scores, indicating impaired HRQL. Chronic PA infection was significantly associated with the severity of bronchiectasis.

## Background

The incidence of lung disease caused by nontuberculous mycobacteria (NTM) has increased worldwide [1, 2]. *Mycobacterium avium* complex (MAC), the most common cause of NTM in Japan, generally causes chronic, slowly progressive lung disease in middle-aged men with risk factors such as smoking and structural lung diseases or in nonsmoking women without any of these risk factors [3]. Antimicrobial therapy for MAC lung disease (MACLD) for >12 months yields a success rate of approximately 75% [4], but high rates of recurrence have been reported after discontinuation of treatment [5]. Further studies have shown that the use of multiple antimicrobials is associated with multiple potential adverse effects, including ethambutol-induced ocular toxicity [3]. Because of the increasing prevalence and chronicity of MACLD, as well as the limited data regarding antimicrobial therapies associated with potential adverse effects, to monitor the patient’s overall health status, patient-reported outcome measures that represent health-related quality of life (HRQL) have become increasingly important for MACLD [6].

Bronchiectasis is a respiratory disease characterised by permanent dilation of the bronchus and chronic respiratory symptoms such as daily sputum, coughing, shortness of breath, and recurrent respiratory infections [7]. The severity of bronchiectasis has been associated with worse HRQL, as evaluated by St. George’s Respiratory Questionnaire (SGRQ) and Leicester Cough Questionnaire, and with a poor prognosis [8]. In addition to idiopathic causes, the aetiology of bronchiectasis can include factors such as previous severe respiratory infection, primary or secondary immunodeficiency, allergic bronchopulmonary aspergillosis, rheumatic or connective tissue disease, chronic obstructive pulmonary disease (COPD), or an impairment of ciliary clearance such as primary ciliary dyskinesia [9]. NTM has been described as both a cause of bronchiectasis and a secondary feature of established bronchiectasis that results from another aetiology [10]. Notably, most MACLD patients had bronchiectasis in high-resolution computed tomography (HRCT) studies [11, 12].

Previous studies have reported that in bronchiectasis patients, chronic *Pseudomonas aeruginosa* (PA) infection was associated with worse HRQL and with significant increases in disease exacerbation, hospital admission, and patient mortality [13]. Additionally, few studies have investigated the frequencies of or risk factors for chronic PA infection in MACLD patients [14]. Accordingly, little is known about the impact of chronic PA infection on HRQL in MACLD patients. The current study aimed to evaluate HRQL via the 36-item Short-Form health survey (SF-36) and SGRQ to investigate the impact of chronic PA infection on MACLD patients.

## Methods

### Study population

This single-centre cross-sectional study was conducted using the prospective, observational cohort registry at Keio University Hospital with patients aged 20 years or older who had been diagnosed with MACLD (UMIN000007546). MACLD was diagnosed in accordance with the 2007 American Thoracic Society/Infectious Disease Society of America guidelines [3]. Patients with MACLD who completed the HRQL questionnaire between May 2012 and July 2014 were included in the study. The Keio University Hospital ethics review board approved the study protocol (#20110267). All patients provided written informed consent. We followed the Strengthening the Reporting of Observational Studies in Epidemiology (STROBE) statement (http://www.strobe-statement.org/).

### HRQL assessment

For the HRQL assessment, all patients completed the SF-36 version 2 [15] and the SGRQ in Japanese [16]. Both questionnaires have been validated for MACLD [17]. The internal consistencies of the SF-36 and SGRQ were assessed using Cronbach’s α.

The SF-36 included eight subscales: physical functioning, role-physical, bodily pain, general health perception, vitality, social functioning, role-emotional, and mental health. Three summary scores were derived from the eight subscales, which were adjusted for Japanese patients: physical, mental, and role/social component summary scores [18]. The eight subscales ranged from 0 to 100, and the three summary scores were transformed to fit a norm-based score for the general Japanese population, with a mean score of 50 and a standard deviation of 10. Higher scores indicated better HRQL.

The SGRQ included symptoms, activity, and impact components that corresponded with measures of respiratory symptoms, impairment of physical activity, and social and psychological disturbances, respectively. The total score was calculated with the inclusion of each component. Possible scores were derived from the SGRQ range of 0–100, with higher scores indicating worse respiratory HRQL.

### Assessment of clinical parameters

Assessed patient characteristics included age, sex, body mass index (BMI), disease duration, smoking status, underlying pulmonary diseases, Charlson Comorbidity Index (CCI) [19], treatment status, bacterial smear and culture results for MAC, and laboratory data. Treatment status was classified as never treated, previously treated, or currently treated. MAC isolates were identified as previously described [20]. Sputum smear or culture data were defined based on results obtained between 6 months before and 6 months after registration. If patients did not expectorate sputum, then they were recorded as negative. Chronic PA infection was deemed present when PA was isolated from sputum culture on two or more occasions ≥3 months apart during the period of 6 months prior to registration through 6 months after registration [21].

### Assessment of pulmonary function tests and high-resolution computed tomography

Pulmonary function tests (PFTs) and HRCT images were evaluated within 1 week of HRQL assessment. Patients who were in stable condition performed PFTs with an electronic spirometer (Chestac-9800 or HI-801; Chest M.I., Tokyo, Japan) in accordance with American Thoracic Society/European Respiratory Society recommendations.

HRCT images were assessed for the presence of cavitary lesions and severity of bronchiectasis. Two pulmonologists who were blinded to the clinical data performed the evaluations, and consensus review with an additional investigator was utilised to resolve any discrepancies. Bronchiectasis was assessed using a modified Reiff score, as in previous bronchiectasis studies [21]. The number of lobes involved (of a total of six; the lingula was considered separate) and the degree of dilatation (tubular = 1, varicose = 2, and cystic = 3) were calculated (range, 0–18).

### Statistical analyses

All statistical analyses were performed using JMP version 12.0 (SAS Institute Japan Ltd., Tokyo, Japan). Continuous variables were analysed as median and interquartile range (IQR) or number and percentage (%). Fisher’s exact test and the Mann-Whitney test were used for comparisons between two groups, and the Kruskal-Wallis test was used for comparisons between three groups during the analysis of categorical and continuous variables, respectively. To investigate the impact of chronic PA infection on HRQL, we first performed univariate analysis on each of the HRQL scores. An analysis of covariance (ANCOVA), adjusted for age, sex, BMI, CCI, and underlying pulmonary disease, was then performed to test the statistical significance of group differences. All *P*-values <0.05 were considered statistically significant.

## Results

### Patient characteristics and clinical features

A total of 244 patients with MACLD were included in the study. Table 1 shows clinical characteristics of the 244 MACLD patients with and without chronic PA infection. The median age of the patients was 68 years and the IQR was 62–75 years. A total of 196 patients (80%) were women, and the median duration of disease was 5.7 years. The median BMI was 19.5 kg/m^2^, and 220 patients (90%) reported that they had never smoked. A history of pulmonary tuberculosis was the most common underlying pulmonary disease (*n* = 23, 9.4%), followed by asthma (*n* = 8, 3.2%), lung cancer (*n* = 3, 1.2%), COPD (*n* = 2, 0.8%), and interstitial lung disease (*n* = 1, 0.4%). With regard to treatment status of MACLD, 116 (48%) had never been treated, 40 (16%) had been treated previously but were not currently being treated, and 88 (36%) were currently undergoing treatment. Sputum smear test results or cultures obtained 6 months before and 6 months after registration were positive for MAC in 83 (34%) and 138 (57%) patients, respectively. CCI was significantly higher in patients with chronic PA infection than in those without (*P* = 0.048). Patients with chronic infection tended to be older than those without, and they were less likely to yield a positive sputum smear or culture; however, these tendencies were not statistically significant.

**Table 1:** Clinical characteristics of 244 *Mycobacterium avium* complex lung disease patients with and without chronic *Pseudomonas aeruginosa* infection

Characteristic	All patients (*n* = 244)	With chronic infection (*n* = 19)	Without chronic infection (*n* = 225)	*P* value
Age, years	68 (62–75)	74 (67–76)	67 (61–75)	0.055
Sex, female	196 (80)	14 (74)	182 (81)	0.546
Disease duration, years	5.7 (2.3–9.7)	5.3 (2.3–10.3)	5.8 (2.3–9.6)	0.622
Body mass index, kg/m^2^	19.5 (17.5–21.3)	18.8 (17.3–21.2)	19.6 (17.5–21.3)	0.515
Smoking status				1.000
Never	220 (90)	17 (89)	202 (90)	
Former	25 (10)	2 (11)	23 (10)	
Current	0 (0)	0 (0)	0 (0)	
Charlson Comorbidity Index	0 (0–1)	1 (1–1)	0 (0–1)	0.048
Underlying pulmonary disease				
History of pulmonary TB	23 (9)	4 (21)	19 (8)	0.089
Asthma	8 (3)	1 (1)	7 (3)	0.482
COPD	2 (1)	0 (0)	2 (1)	1.000
Lung cancer	3 (1)	1 (1)	2 (1)	1.000
Interstitial lung disease	1 (0.4)	0 (0)	1 (0.4)	1.000
Treatment status				0.452
Never	116 (48)	10 (53)	106 (47)	
Previous	40 (16)	1 (5)	39 (17)	
Current	88 (36)	8 (42)	80 (36)	
Bacterial status^a^				
Positive smear	83 (34)	3 (15)	80 (36)	0.083
Positive culture	138 (57)	7 (37)	131 (59)	0.090

Data show the median (interquartile range) or number (%) of patients

TB, tuberculosis; COPD, chronic obstructive pulmonary disease

^a^Bacterial status for mycobacterium

### PFT results, HRCT findings, and laboratory data

**Fig. 1:**
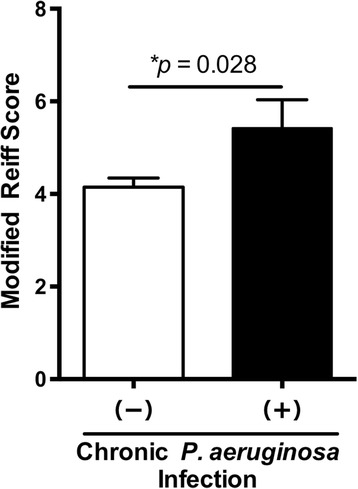
Modified Reiff scores associated with the presence or absence of chronic *Pseudomonas aeruginosa* infection. Modified Reiff scores were higher in the presence of chronic *P. aeruginosa* infection

Table 2 shows the results of PFTs, HRCT findings, and laboratory data in our cohort of MACLD patients. The median values of PFT results were within normal ranges, and there were no significant differences between patients with and without chronic PA infection. There was weak evidence that forced expiratory volume in the first second was lower in patients with chronic infection (*P* = 0.079). During the HRCT analyses, the incidence of cavitary lesions did not differ significantly between the two groups. Almost all patients exhibited bronchiectasis, and the modified Reiff score was higher for patients with chronic infection than for those without (Fig. 1). During laboratory analyses, white blood cell counts, haemoglobin, and C-reactive protein (CRP) were within normal ranges. However, haemoglobin was significantly lower (*P* = 0.049) in patients with chronic PA infection compared with those without; in contrast, CRP was significantly higher (*P* = 0.005) in patients with chronic PA infection.

**Table 2:** Pulmonary function test results, high-resolution computed tomography findings and laboratory data in 244 *Mycobacterium avium* complex lung disease patients with and without chronic *Pseudomonas aeruginosa* infection

	All patients (*n* = 244)	With chronic infection (*n* = 19)	Without chronic infection (*n* = 225)	*P* value
PFT	(*n* = 243)	(*n* = 19)	(*n* = 224)	
%FVC	95 (81–108)	94 (75–102)	96 (82–109)	0.234
%FEV_1_	87 (73–99)	81 (66–98)	88 (74–100)	0.079
HRCT findings				
Cavitary lesion	59 (24)	4 (21)	55 (24)	1.000
Presence of bronchiectasis	233 (95)	19 (100)	214 (95)	0.609
Modified Reiff score	4 (2–6)	5 (3–7)	3 (2–6)	0.028
Laboratory data				
WBC/μL	5250 (4500–6375)	5700 (4700–7400)	5200 (4500–6200)	0.209
Haemoglobin, g/dL	13 (12–14)	12 (11–13)	13 (12–14)	0.049
CRP, mg/dL	0.1 (0.0–0.3)	0.3 (0.1–1.9)	0.1 (0.0–0.3)	0.005

Data show the median (interquartile range) or number (%) of patients

*PFT*, pulmonary function test; *FVC*, forced vital capacity; *FEV*_1_, forced expiratory volume in the first second; *HRCT*, high-resolution computed tomography; *WBC*, white blood cell; *CRP*, C-reactive protein

### SF-36 and SGRQ

Table 3 shows the results of SF-36 and SGRQ in our cohort of MACLD patients. The internal consistency of these scores was good (Cronbach’s α coefficients: SF-36, 0.89–0.91; SGRQ 0.91–0.95). With regard to SF-36 subscales, the physical functioning scores were significantly lower in patients exhibiting chronic PA infection compared with those patients without chronic PA infection (*P* = 0.029). Nearly all of the SGRQ scores were significantly higher in patients exhibiting chronic infection compared with those without (*P*-values: symptom, 0.008; impact, 0.006; total, 0.012); notably, the activity scores were not significantly different between groups.

**Table 3:** Health-related quality of life in the 244 *Mycobacterium avium* complex lung disease patients with and without chronic *Pseudomonas aeruginosa* infection

	All patients (*n* = 244)	With chronic infection (*n* = 19)	Without chronic infection (*n* = 225)	*P* value
SF-36				
Physical functioning	90 (75–95)	85 (69–90)	90 (78–95)	0.029
Role-physical	88 (63–100)	75 (56–88)	88 (63–100)	0.057
Bodily pain	74 (52–100)	74 (41–100)	74 (52–100)	0.269
General health	52 (40–62)	47 (40–52)	52 (40–65)	0.292
Vitality	63 (44–75)	56 (50–75)	63 (44–75)	0.654
Social functioning	88 (63–100)	75 (38–100)	88 (63–100)	0.135
Role-emotional	92 (58–100)	92 (58–100)	92 (58–100)	0.926
Mental health	75 (60–85)	70 (55–80)	75 (60–85)	0.461
PCS	48 (40–54)	40 (36–48)	48 (40–54)	0.071
MCS	50 (44–56)	49 (43–55)	51 (44–46)	0.702
RCS	51 (39–56)	50 (34–55)	51 (40–57)	0.451
SGRQ				
Symptom	30 (16–48)	48 (30–52)	30 (14–46)	0.008
Activity	24 (6–46)	35 (19–48)	23 (6–46)	0.141
Impact	12 (4–31)	27 (17–42)	11 (3–30)	0.006
Total	20 (8–36)	30 (23–49)	18 (8–36)	0.012

Data show the median (interquartile range) or number (%) of patients

*SF-36*, 36-item Short-Form health survey; *PCS*, physical component summary; *MCS*, mental component summary; *RCS*, role/social component summary; *SGRQ*, St. George’s Respiratory Questionnaire

### Comparisons of physical component subscale score and SGRQ based on nominal variables using ANCOVA

**Fig. 2:**
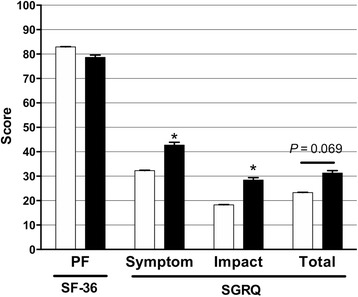
Comparisons of physical functioning (PF) subscale scores derived from the 36-item Short Form health survey (SF-36) and symptoms, impact, and total (excluding activity score) scores derived from the St. George’s Respiratory Questionnaire (SGRQ) presented as least square means based on the presence or absence of chronic *Pseudomonas aeruginosa* infection after adjustment for age, sex, body mass index, Charlson comorbidity index, and underlying pulmonary disease in *Mycobacterium avium* complex lung disease patients

There was no significant difference in physical component subscale score between patients with and without chronic infection (*P* = 0.303). However, SGRQ symptoms and impact scores were significantly higher in patients exhibiting chronic infection (symptoms, *P* = 0.043; impact, *P* = 0.021). There was weak evidence indicating that total SGRQ scores were higher for patients with chronic infection (*P* = 0.069) (Fig. 2).

### Clinical characteristics associated with chronic PA infection in MACLD patients

Table 4 shows the clinical characteristics associated with chronic PA infection in the subset of 19 MACLD patients who exhibited signs of this illness. Other underlying diseases included rheumatoid arthritis (*n* = 2), mixed connective tissue disease (*n* = 1), gastroesophageal reflux disease (*n* = 1), liver cirrhosis (*n* = 1), and chronic sinusitis (*n* = 1). During PFTs, six patients exhibited restrictive dysfunction and six exhibited obstructive dysfunction. Ten patients (53%) provided sputum cultures and smears that were negative for NTM. With regard to treatment status for MACLD, 10 (53%) patients had never been treated, one (5%) had been treated in the past but was not currently being treated, and eight (42%) were currently undergoing treatment. Four patients provided sputum cultures and smears that were negative for NTM, despite never having been treated for MACLD. For patients who had previously received or were currently receiving multiple antimicrobial therapy, the median treatment duration was 4.3 years (IQR, 2.9–10.1 years).

**Table 4:** Clinical characteristics of the 19 *Mycobacterium avium* complex lung disease patients with chronic *Pseudomonas aeruginosa* infection

No.	Age	Sex	BMI	CCI	Underlyingdisease	MACLD duration years	Hb	CRP	%FVC	%FEV_1_	Sputumsmear/ culture^a^	Cavity	Modified Reiff score	Treatment status for MACLD	Treatment regimen^b^	Treatment duration, years
1	74	F	17.3	0		10.3	13.1	1.1	99	86	- / -	+	7	Current	CAM + RFP	8.8
2	76	F	16.2	1	Old pTB, GERD	2.3	12.1	0.0	95	86	- / +	–	3	Never		
3	75	M	20.2	1	LC post-lobectomy	3.2	13.8	0.5	71	55	+ / +	–	4	Current	CAM + EB + RFP	3.4
4	76	F	17.3	1	MCTD	2.1	11.0	0.2	94	114	- / +	–	5	Never		
5	57	F	18.8	1	RA	5.3	11.7	4.2	68	54	- / -	–	4	Current	CAM + EB + RFP	5.2
6	69	F	26.1	0		15.9	13.1	0.3	78	68	- / -	+	7	Previous	CAM + RFP + EB + KM	4.3
7	77	M	21.4	1	Old pTB, ABPM	6.5	9.8	0.2	116	105	- / -	–	3	Never		
8	77	F	14.9	1		1.9	12.2	0.0	98	102	+ / +	–	7	Never		
9	67	M	18.5	0	Old pTB	2.3	14.6	0.1	112	83	- / -	–	1	Current	CAM + EB + RFP	2.3
10	74	F	19.5	0		13.0	13.5	1.2	87	75	- / -	–	10	Current	CAM + EB + RFP	11.4
11	81	F	18.4	0		14.8	11.0	0.3	103	102	+ / +	–	7	Never		
12	67	F	21.2	1	RA	3.3	13.0	2.4	98	71	- / -	–	6	Never		
13	73	F	18.5	0		9.7	10.5	5.4	80	82	- / -	+	11	Never		
14	71	M	21.9	2	Liver cirrhosis	5.1	15.0	0.1	102	82	- / +	–	6	Never		
15	69	M	16.9	0	Old pTB	6.5	12.7	1.9	58	65	- / +	+	8	Current	CAM + EB + RFP	1.7
16	77	F	20.9	1		9.5	12.0	5.0	92	70	- / -	–	2	Never		
17	63	F	16.8	1		4.3	11.7	0.2	69	64	- / +	–	5	Current	CAM + RFP	4.1
18	74	F	21.4	1		0.6	13.4	0.0	106	98	- / +	–	2	Never		
19	35	F	18.9	0	Chronic sinusitis	14.5	10.7	0.0	75	66	- / -	–	5	Current	AZM + RFP + EB	13.4

*BMI*, body mass index; *CCI*, Charlson comorbidity index; *MACLD*, *Mycobacterium avium* complex lung disease; *Hb*, haemoglobin; *CRP*, C-reactive protein; *FVC*, forced vital capacity; *FEV*_1_, forced expiratory volume in the first second; *CAM*, clarithromycin; *RFP*, rifampicin; *pTB*, pulmonary tuberculosis; *GERD*, gastroesophageal reflux disease; *LC*, lung cancer; *EB*, ethambutol; *MCTD*, mixed connective tissue disease; *RA*, rheumatoid arthritis; *KM*, kanamycin; *ABPM*, allergic bronchopulmonary mycosis; *AZM*, azithromycin;

^a^Sputum smear or culture for mycobacterium. ^b^Treatment regimen included multiple antimicrobial therapy for MACLD

## Discussion

To our knowledge, this is the first study to evaluate the effects of chronic PA infection on HRQL, as determined by the SF-36 and SGRQ, using only MACLD patients; many of these MACLD patients exhibited bronchiectasis. Our results showed that chronic PA infection reduced HRQL as determined by the SGRQ. The severity of bronchiectasis in HRCT, but not the presence of cavitary lesions, was associated with chronic PA infection in MACLD patients. We also determined clinical characteristics associated with treatment and bacterial infection status in these patients.

In the present study, chronic PA infection worsened SGRQ scores of MACLD patients, which is consistent with previous studies wherein bacterial infection affected HRQL in COPD and bronchiectasis patients [22–25]. In COPD patients, bacterial colonisation (particularly *Haemophilus influenza*) and total bacterial load were the primary factors associated with increased daily symptoms and poorer SGRQ scores [22–24]. In studies of bronchiectasis, SGRQ-determined HRQL was significantly worse in patients with chronic PA infection compared to other patient groups [21, 25]. Although there was a small number of patients with chronic PA infection in the current study, our results suggest that chronic infection may affect the physical functioning and role-physical subscales of the SF-36, as well as the physical component summary. The mechanisms by which chronic PA infection influences HRQL are unclear; however, bronchiectasis patients with chronic PA infection exhibit higher cytokine production [26, 27], which may have contributed to the higher CR*P* values observed in the current study.

Severity of bronchiectasis, rather than the presence of cavitary lesions, was significantly associated with the presence of chronic PA infection in MACLD patients during this study. This association is consistent with previous reports regarding chronic PA infection in bronchiectasis patients [28]. An Italian study of a cohort of bronchiectasis patients indicated that NTM lung disease produced cylindrical bronchiectasis, a disease of lesser severity, and fewer pulmonary exacerbations than chronic PA infection [29]. In addition to bronchiectasis, MACLD may manifest as various types of lesions, including infiltrates, nodules, and cavitary lesions. Notably, cavitary lesions have been associated with reduced pulmonary function and lower HRQL, and they are predictive of disease progression requiring treatment and an ultimately poor prognosis [12, 30, 31]. Moreover, cavitary lesions have been revealed as an independent risk factor for MACLD that may develop into chronic pulmonary aspergillosis [32], because *Aspergillus* colonises both the airway and cavitary lesions. It has also been reported that bronchiectasis is associated with lower pulmonary function and is predictive of disease progression requiring treatment, despite its unknown impact on prognosis [12, 33]. Although it remains unclear whether chronic PA infection is the cause or result of advanced disease, our current study indicates that chronic PA infection is associated with bronchiectasis, in contrast to the *Aspergillus* colonisation that is associated with both bronchiectasis and cavitary lesions.

The nine patients who were either undergoing treatment for MACLD or had previously undergone treatment for MACLD received long-term antimicrobial therapy during this study; however, 10 patients exhibited chronic PA infection despite no history of treatment for MACLD. Moreover, four out of 10 patients exhibited negative results during analysis of both sputum smears and cultures. Previous studies indicated that NTM-infected bronchiectasis patients, including those with cystic fibrosis, had a lower rate of chronic PA infection compared with NTM-uninfected patients [34–36]. In the present study, MACLD patients with chronic PA infection exhibited a lower rate of NTM-positive sputum cultures, regardless of similar treatment status. Although the parameters involved in the interaction between PA and NTM in the lung are unknown, these results may reflect a dominant pathogenic species, as has been suggested in previous studies [34–36]. Furthermore, it was recently reported that almost half of untreated patients with stable MACLD exhibited spontaneous conversion [37]. Therefore, bronchiectasis patients with chronic PA infection (that is predictive of a poor prognosis) may include post-MACLD patients.

The current study had several potential limitations. First, the study design was cross-sectional; therefore, it was difficult to determine causal associations, particularly with regard to the influence of treatment or the presence of MAC on bronchiectasis. Moreover, we could not identify a risk factor for or assess the impact of chronic PA infection on prognosis. Second, the method and frequency of sputum smear/culture testing for PA were dependent on each attending physician rather than an established protocol. Furthermore, in our study, we used the most stringent definition of chronic infection that was used in previous studies [13], resulting in a very small number of patients who were classified as having chronic PA infection. Although these biases may have underestimated chronic PA infection, we identified impaired HRQL associated with chronic PA infection. Third, we did not include patients who exhibited chronic PA infection without NTM infection. Although these patients are an important control group, their exact classification might be difficult due to the spontaneous conversion of MACLD [37]. Finally, we did not identify any factors associated with pulmonary physicians’ decisions regarding the initiation, continuation, or discontinuation of antimicrobial therapy for MACLD or chronic PA infection. However, the indications for treatment, optimal durations of treatment for patients with MACLD, and management approaches for chronic PA infection were unclear in this study. Further studies that include MACLD patients with chronic PA infection are needed to investigate the relationships between antimicrobial treatment and patient outcomes. Additionally, to clarify risk factors and the influence of chronic PA infection on prognosis, prospective observational multicentre studies are needed that incorporate microbiological data and imaging, especially those involving newly diagnosed MACLD and bronchiectasis patients.

## Conclusions

MACLD patients with chronic PA infection exhibited a significantly lower HRQL than those without, as indicated by higher SGRQ scores. Chronic PA infection was significantly associated with the severity of bronchiectasis.

## Abbreviations

ANCOVA: Analyses of covariance
BMI: Body mass index
CCI: Charlson comorbidity index
CF: Cystic fibrosis
COPD: Chronic obstructive pulmonary disease
CRP: C-reactive protein
HRCT: High-resolution computed tomography
HRQL: Health-related quality of life
IQR: Interquartile range
MAC: *Mycobacterium avium* complex
MACLD: *Mycobacterium avium* complex lung disease
NTM: Nontuberculous mycobacteria
PA: *Pseudomonas aeruginosa*
PFTs: Pulmonary function tests
SF-36: 36-item Short-Form health survey
SGRQ: St. George’s Respiratory Questionnaire
TB: Tuberculosis
